# A Landscape of CRISPR/Cas Technique for Emerging Viral Disease Diagnostics and Therapeutics: Progress and Prospects

**DOI:** 10.3390/pathogens12010056

**Published:** 2022-12-29

**Authors:** Shyam Tripathi, Purnima Khatri, Zeeshan Fatima, Ramendra Pati Pandey, Saif Hameed

**Affiliations:** 1 Centre for Drug Design Discovery and Development (C4D), SRM University, Delhi-NCR, Rajiv Gandhi Education City, Sonepat 131029, India; 2 Department of Microbiology, SRM University, Delhi-NCR, Rajiv Gandhi Education City, Sonepat 131029, India; 3 Department of Medical Laboratory Sciences, College of Applied Medical Sciences, University of Bisha, Bisha 61922, Saudi Arabia; 4 Amity Institute of Biotechnology, Amity University Haryana, Gurugram 122413, India

**Keywords:** emerging infectious viruses, CRISPR Cas9/based diagnosis, gene-editing, disease treatment

## Abstract

Viral diseases have emerged as a serious threat to humanity and as a leading cause of morbidity worldwide. Many viral diagnostic methods and antiviral therapies have been developed over time, but we are still a long way from treating certain infections caused by viruses. Acquired immunodeficiency syndrome (AIDS) is one of the challenges where current medical science advancements fall short. As a result, new diagnostic and treatment options are desperately needed. The CRISPR/Cas9 system has recently been proposed as a potential therapeutic approach for viral disease treatment. CRISPR/Cas9 is a specialised, effective, and adaptive gene-editing technique that can be used to modify, delete, or correct specific DNA sequences. It has evolved into an advanced, configurable nuclease-based single or multiple gene-editing tool with a wide range of applications. It is widely preferred simply because its operational procedures are simple, inexpensive, and extremely efficient. Exploration of infectious virus genomes is required for a comprehensive study of infectious viruses. Herein, we have discussed the historical timeline-based advancement of CRISPR, CRISPR/Cas9 as a gene-editing technology, the structure of CRISPR, and CRISPR as a diagnostic tool for studying emerging viral infections. Additionally, utilizing CRISPR/Cas9 technology to fight viral infections in plants, CRISPR-based diagnostics of viruses, pros, and cons, and bioethical issues of CRISPR/Cas9-based genomic modification are discussed.

## 1. Introduction

CRISPRs (clustered regularly interspaced short palindromic repeats) have been found in a wide variety of prokaryotes, including the majority of Archaea and many eubacteria. They are composed of a series of 24–47 bp repeating sequences commonly referred to as direct repeats (DR), separated by unique sequences of equal length (spacers) [1,2,3,4]. The origin of the spacers is still unknown, but some recent studies have identified some of them as bits of foreign DNA, most of which are viral in nature [5,6].

CRISPR stores sequence information about harmful mobile genetic elements in an array and then uses that information to perform targeted degradation of DNA or RNA, depending on the CRISPR type [7,8]. Each CRISPR array consists of a set of direct repetitions that are spaced out by brief sequences called “spacers” that match DNA from earlier invaders [6]. Ishino et al. [9] performed the first examinations of CRISPR 29 bp repeats in 1987 in *Escherichia coli* [10]. The size and sequence of repetitions in a single CRISPR array are constant [11,12]. A new path for gene rehabilitation became available in biomedical research in 2013 as a result of the success of genome changes made possible by the CRISPR/Cas9 tool in cultured human cells [13,14]. Using restriction enzyme “nucleases”, site-specific DNA divisions are inserted, and then, DNA repair mechanisms are used to close the DNA breaks. This is referred to as gene editing [15].

Exogenous DNA double-strand breaks in genomes can be caused by a variety of genome engineering operating systems, including mega nuclease, zinc-finger nucleases (ZFNs), transcription activator-like effector nucleases (TALENs), and CRISPR/Cas nuclease systems [16]. After that, the homology-directed repair (HDR) pathway with repair templates or the nonhomologous end joining (NHEJ) pathway without repair templates are used to complete cell DNA repairs that were initiated by DNA lesions [17]. Currently, the CRISPR/Cas genome editing system has been developed as a trustworthy tool for targeted gene alterations in a wide range of animal species, including gut microbiota [18], and invasive viruses, which cause changes in host–virus relationships. As scientific interest in gene editing research grows, a new branch of medicine based on CRISPR/Cas9 editing technology is entering the clinical stage for the treatment of viral infections [19].

The CRISPR/Cas system acts in a sequence-specific manner by recognizing and cleaving foreign DNA or RNA. The defence system has three stages: stage I, adaptation or spacer acquisition; stage II, crRNA synthesis; and stage III, target interference [7]. A separate protospacer sequence from the invasive mobile genetic element is integrated into the CRISPR array in the first stage, creating a new spacer [20]. This process exhibits the immune system’s adaptability and enables the host organism to memorise the genetic material of the invader [21]. The CRISPR array is translated into a lengthy precursor crRNA (pre-crRNA) that is processed into mature guide crRNAs that carry the invaders’ stored sequences to enable immunisation. In the last, i.e., interference step, of immunity, mature cr-RNAs serve as guides to precisely interfere with invasive nucleic acids [22]. The effector module—either another Cas protein complex or a single large protein—is guided by a crRNA to recognize and cleave target DNA (or in some cases, RNA).

Based on crRNA processing and subsequent action, CRISPR/Cas technologies are classified into three different categories [23]. Type 1 CRISPR/Cas classifications use Cas5 or Cas6 for crRNA pre-processing; Cas3, Cascade, and crRNA are required for extra fragmentation for intervention [23]. In the type 2 CRISPR/Cas system, RNase III, trans-activating RNA (tracrRNA), and an undiscovered protein component are all involved in trimming the 5′ ends of the target DNA, although Cas9 typically functions under the guidance of crRNA to target DNA [23]. 

In the type 3 CRISPR/Cas system, Cas6 is employed in the same manner as the type 1 system to carry out crRNA 3′ end trimming. The pointing of RNA by this approach, which is carried out by a specific composite known as “the type III Csm/Cmr complex”, makes it unique [23]. Whereas the majority of research on CRISPR/Cas systems emphasizes its primary function as a defence against invasive heritable characters, its immersion in many biological developments, such as virulence regulation, genome evolution, and DNA repair, is increasingly strong [23]. It has been shown that the *E. coli* Cas1 protein can break down replication forks, 5′ flaps, and single-stranded and branching DNA species. Moreover, Cas1 interacts with RecB, RecC, and RuvB [24], which point to a potential function in DNA repair but also enhance spacer acquisition through the RecBCD complex [25]. Additionally, it has been noted that “CRISPR/Cas” is activated by the buildup of misfolded proteins in the membrane of *E. coli* [25], indicating a potential function in managing the buildup of faulty proteins [26].

Viruses cause a wide range of acute and chronic disorders, some of which can progress to life-threatening conditions, such as the current coronavirus disease 2019 (COVID-19) pandemic. Some of them, such as herpes simplex viruses, only cause minor illnesses [14]. Major viral infectious diseases, including the human immunodeficiency virus (HIV), hepatitis B virus (HBV), and human papillomavirus (HPV) are currently putting human health and global security in danger [27]. They undoubtedly increase the social pressure on international welfare organisations [28]. In comparison to other infectious viruses such as the “herpes simplex virus”, the three viruses listed above are more dangerous to humans. Due to reduced success rates with medical treatment, they are also harder to treat once contracted [29]. Treatment of viral infections is challenging due to viruses’ excessive demand for cellular resources and the emergence of dormant virus-related pools in the hosts. Moreover, many human viruses can develop mutant strains that can spread and even move between several classes, leading to pandemics [30]. As a result, a series of antiviral strategies have been developed, including genetically engineered drugs, herbal cures, medicines obtained from animals, synthetic pharmaceuticals, and therapies based on antibodies [31,32,33,34,35].

There are several approaches with enormous potential for combating harmful human pathogenic viruses, but the CRISPR/Cas genome editing strategy stands out as a paradigm-shifting advance in biomedicine and gene therapy [14].

## 2. The Revolution by CRISPR/Cas Techniques

With the practice of a brief gRNA and the Cas9 protein, gene editing technology known as CRISPR/Cas9 can change virtually any targeted genomic area. When compared to other outdated gene editing methods, CRISPR/Cas9 technology is speedier and more proficient. The five direct repeats of a 24-nucleotide-long repetitive sequence were found in 1987 when the gene for converting alkaline phosphatase isozyme in *E.coli* was discovered. CRISPR was initially reported as a result of this research, which was widely publicized between 1993 and 2005 [9]. The roles of CRISPR were established in 2007 following the finding of genes around the non-native viral DNA sequences found in CRISPR spacers in 2005 and the CRISPR locus in 2002. To make CRISPR the most potent gene-editing tool now accessible, two labs designed it at the same time in 2013. The first clinical application of CRISPR/Cas9 for the cure of lung cancer took place in China in 2016. Several clinical treatments based on CRISPR/Cas9 technologies have been reported in the last three years. The year 2020 saw the awarding of the Nobel Prize to J. Doudna and E. Charpentier for their discoveries using CRISPR/Cas9 technology [36]. The entire journey from 1987 to today for our ease of study can be divided into the following phases (Table 1):

Identification phase: 1987–2010;

Application phase: 2011–ongoing [37].

**Table 1:** The progression of the CRISPR/Cas systems in research and applications over time.

Phases	Year	Revolutionary Events	References
Identification phase	1987	The genome of the bacteria *E. coli* was where short direct repeats were initially discovered.	[10]
	1989	First application of gene transfer in humans.	[38]
	1990	First gene delivery for therapeutic intent in ADA-SCID patients.	
	1993	CRISPR locus described.	[39]
	2000	Other bacteria and archaea were found to contain the repetitive sequence that Ishino first discovered in *E. coli*. Gene therapy induced leukaemia in ADA-SCID patients.	
	2002	Identification of the Cas gene and the proposed name for CRISPR. CRISPR transcript was discovered.	
	2003	The first information of investigational discovery of a protein related to CRISPR repeats.	
	2005	Identification of the plasmid and viral sources of spacers. The suggestion that bacteria’s CRISPR/Cas defence mechanism might be adaptive. Recognition of a protospacer-related motif (PAM).	
	2007	*S. thermophilus* provides the first experimental confirmation for the CRISPR adaptive immune system.	[40]
	2008	Recognition of mature CRISPR RNAs (crRNAs) in *E. coli* as anti-phage defence complex guides with Cas proteins. Researching the *Staphylococcus epidermidis* Type III (Csm) CRISPR/Cas interference activity.	[41]
	2009	CRISPR/Cas systems are used to research Pyrococcus furiosus’s antiviral capabilities. Identification of the ssRNA-cleaving type III-B Cmr complex.	[42]
	2010	Determination of the CRISPR/Cas bacterial immunity cleavage at a position 3 nucleotides before the PAM sequences. CRISPR/Cas 9 is recognised as the prokaryotic adaptive immune system.	[23]
Application phase	2011	Classification of the three main category types 1, 2, and 3 of CRISPR/Cas systems. Trans activating CRISPR RNA was discovered (tracrRNA). Using the CRISPR/Cas system from *S. thermophilus* type II in *E. coli* and broadcasting that it is functional in several far-related organisms.	[43]
	2012	The modification of the type2 “CRISPR” system (developed by S. pyogenes) for use in cells of mammalian origin. The primary in vitro example of targeting DNA cleavage by CRISPR. A simplification-focused sgRNA construct.	[44]
	2013	Successfully alteration of the genome in eukaryotic cells using Cas9.	[45]
	2014	Cas, apo/Cas9, guide RNA, and target RNA were isolated as crystal structures.	[46,47]
	2015	Chimeric Cmr complex crystal structure; use of CRISPR/Cas9 in humanoid embryo. Scientists used a technique to fix the HBB locus, which when it is mutated, causes thalassemia blood diseases. Due to its off-target behaviours and inability to forecast the results of gene editing using pre-implantation genetic diagnostic (PGD), the experiment was ineffective.	[48]
	2016	Target recognition and CRISPR/Cas9 nuclear dynamics were discovered in live cells. The NIH gave its clearance to the first CRISPR gene editing human trial. Oncogenic mutations were rendered inactive using CRISPR/Cas9.	[49]
	2017	The discovery of a particular CRISPR protein (CRISPR/Cas 13) that prefers to target RNA to DNA. Creating CRISPR–gold technology, a highly effective CRISPR/Cas9 variant that uses gold nanoparticles to transport the gene-editing tool to cells. The first time CRISPR/Cas9 was used to correct globin gene mutations in human embryos. The first human embryo with CRISPR germline editing was implanted. Initial HIV-1 therapy CRISPR clinical trial.	[50]
	2018	Discovered previously present immunoglobulins that target the Cas9 protein. The likelihood that immune responses could render CRISPR/Cas9 gene therapy ineffective. The first CRISPR-based cancer immunotherapy clinical trial.	
	2019	The first in vivo CRISPR clinical trials for the management of blindness in the “United States” Human cells with Cas12a orthologs showing editing abilities. Several brand-new subtypes of the Type5 CRISPR system are recognized. Cas12k was found to be an RNA-mediated, site-specific incorporation system in *E. coli*. Several Class 1 CRISPR effectors are used; the Fok I domain is combined.	[51]
	2020	Nobel Prize for CRISPR/Cas9 genome editing.	[52]

## 3. CRISPR/Cas9 Gene-Editing Technology

Among the most recent changes in the realm of genome engineering is CRISPR/Cas9 [53]. This method is among the quickest ways to assist scientists in familiarizing with a DNA segment of an organism. This method of gene editing is taken from the CRISPR family of genes found in bacteria, which enables those organisms to recognize viruses and develop defence mechanisms against them [54]. However, it is highly advised to use this approach to examine the function of the alien organism or DNA that exists within the cells. The virus can be detected by microorganisms with such a genome, and this procedure can be carried out in vitro in the lab. Using a tracrRNA molecule that serves as a pilot molecule enables the organism to use the nuclease of Cas9 to persuade a site-focused double-fibre DNA break. The nuclease of the Cas9 performs the role of a DNA strand breaker, allowing double-stranded DNA to be broken [55].

### 3.1. Structure of CRISPR

A high degree of accuracy and very straightforward construction are provided by CRISPR/Cas9. It is dependent on the mark order and the “protospacer adjacent motif (PAM)” sequence for its specificity [56]. The target sequence for each CRISPR locus in the crRNA array is 20 bases long. Typical crRNA arrays have a variety of distinct target sequences [57]. By using the gene arrangement to form bonds with the arrangement of host DNA sequences, Cas9 proteins choose the proper site on the host’s genome. The sequence can be altered and independently produced because it is not a component of the Cas9 protein [58]. The PAM pattern on the coding region is targeted by Cas9. It is difficult to change Cas9 to identify a different PAM sequence [56]. The PAM region for SpCas9, for example, is 5′-NGG-3′ and occurs around every 8 to 12 base pairs in the human genome, and this is ultimately not too restrictive [56]. It is also often a fairly brief and generic pattern that occurs repeatedly at numerous locations throughout the genome. Cas9 uses the crRNA to locate the correct sequence in the host region of DNA after being assembled into a plasmid and transferred into cells. Depending on the Cas9 variant, the Cas9 protein generates either a single-stranded break or a double-stranded break at the required place in the DNA [56]. Host DNA can undergo homology-directed repair in response to appropriately spaced single-stranded breaks. It avoids the non-homologous-end merging that has flowability and a double-stranded break that is less faulty [59]. The intended outcome is for the new sequence to be incorporated into the genome by the cell’s native homology-directed repair HDR process, which will use the given repair template. This new-fangled arrangement is now a part of the cell’s hereditary makeup and is passed on to the cell’s daughter cells after being incorporated [56].

### 3.2. Working Principle

**Figure 1:**
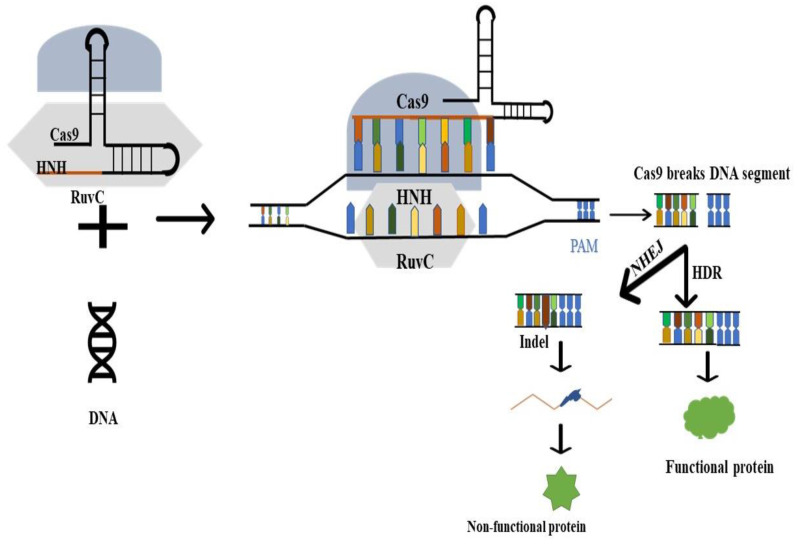
Working principle of CRISPR/Cas9 genome editing technology.

The most widely used CRISPR/Cas system is Type II CRISPR/Cas9 (Figure 1) [60]. The Cas9 protein predominantly recognizes and binds to foreign nucleic acids through Watson–Crick complementary bases between its guide RNA and the foreign DNA with a short PAM [61]. The Cas9 protein’s two nuclease domains, HNH and RuvC, correspondingly split the target sequence site at complementary and non-complementary DNA strands, once they have bound to the foreign DNA [62]. The double-strand DNA breaks (DSBs) caused by this form of cleavage often involve cuts to each of the foreign DNA’s strands and can be fixed using either the NHEJ or HDR pathways [62]. By adding a donor template, the latter repair mechanism can be used to produce the necessary changes in the target gene. Therefore, theoretically, the CRISPR/Cas9 system can modify any target DNA location containing a PAM pattern by changing the guide RNA sequence [63].

### 3.3. Transcriptional Regulations

The CRISPR/Cas9 technique is distinctive in that it is precise and quickly reprogrammable depending on the layout of the experiment. Specific genomic loci may be activated or interfered with by the CRISPR/Cas9 ribonucleoprotein association (CRISPRa or CRIPRi).

**Figure 2:**
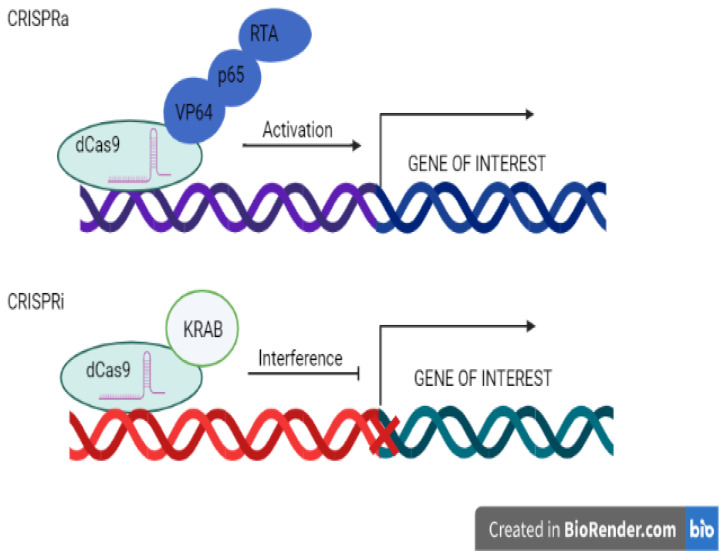
Overview of CRISPR activation (CRISPRa) and CRISPR interference (CRISPRi).

CRISPR activation, also known as CRISPRa, is a CRISPR variation in which a catalytically numb (d) Cas9 entity is united with a transcriptional effector molecule to alter target gene transcription. On one occasion, the gRNA and effector arm are navigated to the genome’s particular position, dCas9 is not able to cleave, and the effector triggers downstream gene transcription (Figure 2).

CRISPRi, or CRISPR interference, is another type of CRISPR in which a catalytically dead (d) Cas9 is merged with a transcriptional effector to modify target gene transcription. In CRISPRi, the gRNA directed at a definite site in the genetic material together with the effector molecule repress downstream located gene transcription as a substitute for activating it [64].

These approaches merged dCas9 to a transcription-regulatory domain that has been thoroughly studied and directs the complex upstream of the transcription initiation position using pre-made gRNAs. The complex can be directed to particular loci by utilising an inactivated dCas9 protein without cleaving or changing the genomic DNA. The fused transcription-regulatory domains can then attract activator or repressor proteins to change gene transcription when Cas9 binds to the specific DNA sequence [65]. Gene transcription is triggered by the synergistic activation mediator (SAM) mechanism, which causes an increase in transcripts up to 3000. It has been demonstrated that SAM can multiplex the activation of genes and activate up to 10 genes at once. SAM is also able to activate long intergenic non-coding RNAs. SAM can also be used to identify the genes that regulate specific desirable phenotypes in disease models or developmental/differentiation processes by using a genome-wide SAM gRNA library for gain-of-function screening. A total of 70,290 different gRNAs, targeting 23,430 different allelic forms with unique transcription initiation sites are present in the human genomic SAM library [66].

## 4. CRISPR as a Diagnostic Tool Studying Emerging Viral Infections

To amplify and recognize a viral sequence, DNA- and RNA-based diagnostics currently use PCR or isothermal amplification. Nucleic acid-based methods such as quantitative or qualitative PCR (qPCR) or reverse transcriptase qPCR (RT-qPCR) are the gold-standard methods because the design of qPCR assays is simple but requires knowledge of the viral sequence. However, the cost, sample-to-answer time, and personnel and equipment requirements limit widespread deployment. Numerous isothermal amplification methods for virus detection have been developed and implemented in order to eliminate the need for costly thermal cycling equipment. These strategies include nicking enzyme amplification reaction (NEAR) [67], recombinase polymerase amplification (RPA) [68], nucleic acid sequence-based amplification (NASBA) [69], loop-mediated amplification (LAMP) [70], and nucleic acid sequence-based amplification (NASBA). Each strategy involves trade-offs with regard to performance traits such as multiplexibility, readout accessibility, sensitivity, specificity, and testing throughput [70,71,72].

Because CRISPR-based diagnostic tools are very precise and sensitive but do not need expensive laboratory equipment, they can enhance conventional procedures. Because Cas–crRNA complexes are inherently sequence-specific, CRISPR-based technologies can be as specific as PCR. In CRISPR/Cas-based diagnostics, Cas12 and Cas13′s distinctive properties are crucial. The collateral cleavage of Cas13 and Cas12 does not demand temperature cycling [73,74]. Additionally, visual readout-compatible reporters can be used to detect collateral cleavage, negating the requirement for pricey apparatuses such as thermocyclers and fluorescent readers [75]. The adaptability of CRISPR-based detection technologies for detecting viral nucleic acids is highlighted by the fact that they have been built for a broad range of both DNA and RNA viruses and make use of various sample processing and amplification techniques, Cas effector proteins, and readouts.

### 4.1. Virus Detection Using Cas9 

Innovative techniques were created that utilised Cas9′s selectivity for identifying viral genes or differentiating between viral strains before the discovery of Cas effector proteins with collateral cleavage activity. Cas9 cleavage was linked with PCR in a technique known as CARP (Cas9/sgRNA-associated reverse PCR), also referred to as ctPCR, to identify specific viral targets (CRISPR-typing PCR). Using PCR, a specific target sequence including two physically separated Cas9 PAM sites was amplified. Two Cas9 sgRNAs may then be used to target the dsDNA that the initial PCR produced in adequate quantities. Versions 1.0, 2.0, and 3.0 of the ctPCR iteratively improved the reactions needed to detect the presence or absence of the target after Cas9 cleavage.

Through PCR amplification of ligated adapters [76], PCR amplification using reverse primers that only amplified a cleaved and ligated region [77], or qPCR amplification in which the relative efficacy of reactions with and without Cas9 was examined, targets were identified [78]. In addition to ctPCR, NASBA-CRISPR cleavage (NASBACC) was created, which combines NASBA amplification, toe-hold sensors, and Cas9 cleavage. It was necessary to have at least one divergent site that interferes with Cas9′s PAM between the sequences to be distinguished in order to execute NASBACC. If Cas9′s PAM was present in the target, it was able to cleave the toehold sensor binding site, allowing for differentiating signals between the two targets [79].

A few viral strains have been subjected to these Cas9 detection techniques. ctPCR is used to identify human papillomavirus (HPV) genes in various subtypes of HPV16 and HPV18. Additionally, the Zika virus strains from Asia and America were distinguished using NASBACC (ZIKV). These investigations show how versatile Cas9 is for identifying and classifying viruses, but their applicability is constrained by the number of reactions required for either amplification or manipulation of the amplified products.

### 4.2. Cas13- and Cas12-Based Detection Technologies

As a result of the characterization of Cas13 and Cas12′s collateral cleavage activity, a number of user-friendly, CRISPR-based detection techniques with the potential for field deployment and massive scaling have been developed [80].

The technology named SHERLOCK (selective high-sensitivity enzymatic reporter unlocking) was created when the collateral activity of Cas13 was identified [81,82]. Leptotrichia wadei (LwaCas13a) was used in SHERLOCKv1 because it had the best target-activated collateral cleavage activity at the time and was programmable, RNA-guided, and active. The isothermal amplification technique RPA was used to boost the assay’s sensitivity because LwaCas13a alone was unable to identify the vast range of probable virus titres in patient samples [68]. Sherlock needed T7-mediated in vitro transcription of the amplified product because RPA yields a dsDNA product, which prevents Cas13 from detecting it. By introducing a synthetic ssRNA molecule flanked by a quencher and an attached fluorescent dye, the amplified target was detected using the fluorescence signal produced by the associated fluorescent dye.

Soon after, other systems such as DETECTR (DNA endonuclease targeted CRISPR trans reporter) [73] and HOLMES (1-h low-cost multifunctional highly efficient system) began to utilise Cas12 for nucleic acid detection [83]. Because Cas12 can detect the immediate result of amplification reactions, these techniques did not require in vitro transcription following amplification (i.e., dsDNA). An inserted ssDNA-quenched fluorescent reporter molecule was cleaved in trans when Cas12′s collateral cleavage activity was triggered by the detected dsDNA target. Different amplification techniques were used by these two Cas12-based technologies; in their first publications, DETECTR used RPA while HOLMES used PCR. These CRISPR-based detection techniques can be used in a variety of settings, including cancer and human genotyping [84].

### 4.3. Cas13- and Cas12-Based Detection of a Single Virus

Singleplex tests have demonstrated the ability of Cas13- and Cas12-based diagnostics to sensitively identify a variety of viral targets. SHERLOCK’s capacity to recognise small amounts of ZIKV in artificial lentivirus samples at known concentrations and in patient samples with a variety of viral titres was first proven during the Zika epidemic [81]. 

In a collection of 25 patient samples, DETECTR was initially utilised to identify the DNA of HPV16 and HPV18. With the exception of two samples, DETECTR produced concordant results when compared to the gold-standard qPCR [73]. Since then, a growing number of CRISPR-based assays have been created and approved for use with human viruses, including the ones that cause Lassa fever, the Epstein–Barr virus, the Powassan virus, the H7N9 influenza virus, the hantavirus, the Ebola virus, and the Japanese encephalitis virus (JEV) [85,86,87]. CRISPR-based tests could be created for any viral pathogen given enough genomic data due to the adaptability of these platforms [88,89]. Once a new virus’ genomic sequence is known, CRISPR-based detection techniques can be quickly tested and validated for it. The advent of SARS-CoV-2 in late 2019 served as a prime example of this. New assays were being created and posted on social media and preprint servers soon after the first SARS-CoV-2 genomes were released [90,91,92,93], and soon after that, peer-reviewed papers. The DETECTR approach was used to create a SARS-CoV-2 assay, which was then validated on more than 70 patient samples, demonstrating how quickly these assays can be created [90]. Similar to this, a SHERLOCK test with excellent agreement with RT-qPCR was validated on more than 150 patient samples in Thailand [94]. Soon after, many more publications appeared [95,96,97]. The FDA’s emergency use authorisation (EUA) process for CRISPR-based SARS-CoV-2 diagnostics was also facilitated by the COVID-19 pandemic. The first FDA authorizations of a CRISPR-based diagnostic came from Mammoth Biosciences and SHERLOCK Biosciences shortly after these CRISPR-based detection technologies were published, underscoring the future potential of CRISPR-based diagnostics for becoming a standard selection of molecular assays for viral diagnosis [98].

**Figure 3:**
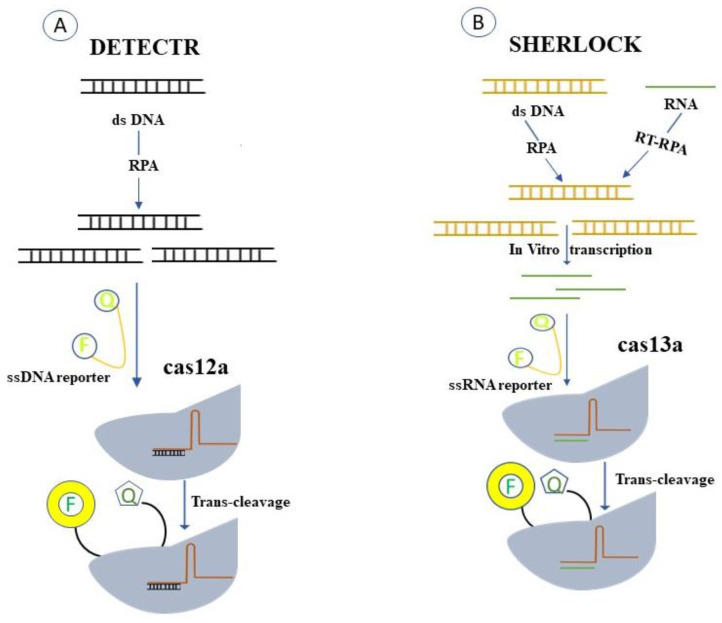
Trans-cleavage activity is used by two exemplary CRISPR-based diagnostic techniques, DETECTR and SHERLOCK. (**A**) Design for DETECTR. The recombinase, single-stranded DNA-binding protein, and strand-displacing polymerase used in RPA, an isothermal alternative to PCR, recognize the target DNA that is amplified by the Cas12a-gRNA complex. When the target is recognized, it breaks apart the nearby ssDNA reporters that are FQ-labelled, restoring the fluorescence. (**B**) SHERLOCK’s design. The target RNA is in vitro transcribed from the amplified DNA by RPA or RT-RPA, and the Cas13a-gRNA complex attaches to it. This initiates the trans-cleavage of the Cas13a-gRNA complex, which cleaves the surrounding FQ-labelled ssRNA reporters.

The field of viral infection is the one where CRISPR-based diagnostic methods have received the greatest attention [99]. The CRISPR/Cas12a and Cas13a families have inspired the development of several research techniques called DETECTR and SHERLOCK, respectively (Figure 3) [100]. In a three-step procedure, DETECTR employs the Type V Cas12a enzyme to connect directly to DNA targets [101]. Usually, a guide RNA drives the Cas12a enzyme to a highly sensitive and specific genome’s double-stranded DNA sequence [73]. Once coupled to its viral genetic target, the Cas12a enzyme indiscriminately cleaves a single-stranded DNA molecule connected to a quencher molecule and a reporter fluorescence [100]. This “collateral” cleavage is recognized by the release of a fluorescent signal from the fluorophore and quencher [73]. The DETECTR method’s main benefit is its great sensitivity, which allows it to identify a single viral particle molecule inside a microliter of the sample [100]. The Type VI Cas13a enzyme is used in the SHERLOCK method to bind and cleave RNA indiscriminately using targets that are crRNAs. When certain sequences are present, target RNA is bound by a target-specific molecule with an attached fluorophore, which then cleaves it collaterally, producing a fluorescence signal that can be recognized and studied to determine the presence of viral nucleic acid [102]. Since its inception, For use in recognizing and diagnosing viruses, SHERLOCK has undergone significant research [75]. Researchers have further improved the approach, creating a more straightforward and focused SHERLOCKv2 protocol [75]. The additional CRISPR-associated Csm6 enzyme was paired with Cas13 enzymes, which more than tripled sensitivity [75]. In both laboratory and clinical settings, viruses can be identified using the DETECTR and SHERLOCK procedures (Figure 3). Although it can be used to diagnose any virus, the DETECTR technique has been widely used to diagnose HPV [102]. Recombinase polymerase amplification (RPA) can enhance highly contagious component multiplication and detection when combined with the SHERLOCK and DETECTR methods. [102]. Additionally, the “SHERLOCK” methodology can be improved for the analysis of HIV, a viral disease that is still a major problem for the entire world [100]. According to HUDSON protocol researchers, universal-flavivirus RPA and crRNAs unique to a particular viral species can both be used to pinpoint conserved sections in these viruses’ genetic material [103]. Although any virus can be detected using SHERLOCK and HUDSON protocols, earlier research concentrated on the detection of flaviviruses such as Dengue, Zika, West Nile, and yellow fever viruses [88,89]. How CRISPR techniques can be used to diagnose the new coronavirus (SARS-CoV-2), an emerging pathogen that has infected over 12.9 million individuals and killed over 500,000 people thus far [104], is of great acute interest to scientists at the moment [22]. The lengthy incubation period is also concerning, as a person with the virus may go up to two weeks without signs before exposure to the disease [105]. In the applications presented, the DETECTR approach has been employed to detect this virus and emphasises determining the occurrence of the N and E gene variations unique to SARS-CoV-2 [106]. If both genes are found, a positive result is produced, and the process has been refined to eliminate false positives brought on by related coronaviruses [93]. Several kits have been created by the CRISPR-associated nucleases Cas9, Cas12, or Cas13, including CASLFA, FELUDA, DETECTR, HOLMES, SHERLOCK, and others [107].

## 5. Utilizing CRISPR/Cas Systems to Fight against Viral Infections

As a dynamic, affordable, practical, and reliable technology, CRISPR/Cas9 is currently demonstrating that it is a game-changing strategy in the fight against emerging viruses. The following are some of the known incidents.

### 5.1. Human Papillomavirus (HPV)

The Papovaviridae family of tiny, double-stranded DNA viruses has about 150 different varieties that have been found thus far [14]. E1–E8 primary viral regulatory proteins, two late capsid proteins, and nine or ten open reading frames (ORFs) are all encoded by the approximately 8 kbp long HPV genome (L1 and L2). Due to their sexual transmission, epithelial tissue tropism, and carcinogenic potential, HPVs have an essential role in human illnesses and public health [108]. Continuous speculative-type HPV infection, such as HPV-16 and HPV-18 [109], is strongly linked to the occurrence of cervical cancer in females [110]. Due to the virus’s capacity to lower activity in a host cell to evade host immune surveillance and the difficulty of removing a viral genome [111] from an infected host cell in a latency state, there is currently no medicine for HPV infection that can achieve a satisfying outcome [112]. Retinoblastoma protein (pRB) and the cellular tumour suppressor p53 is inhibited by the HPV E6 and E7 genes, respectively [113]. Therefore, through the activation of cellular oncogenes, overexpression of E6 or E7 caused by HPVs has a significant likelihood of resulting in the malignant transformation of human cells (e.g., ras or fos) [114]. There are now three HPV vaccines available. The bi- and quadrivalent vaccinations have provided protection against the two most common HPV oncogenic genotypes since 2006 (types 16 and 18) [115]. The year 2014 saw the approval of a nine-valent vaccine that offers defence against five additional cancer-causing HPV strains in addition to types 16 and 18. The male-approved vaccinations quadri- and nine-valent provide defence against the genital wart-causing HPV strains 6 and 11 [116]. In addition to vaccine cost, a major barrier to the acceptance of the HPV vaccine is the lack of experience providing a two-dose vaccine to girls between the ages of 9 and 14 through routine immunisation programmes [117]. Impact studies have shown a considerable reduction in the prevalence of oncogenic and other genotypes present in the immunisation as well as high-grade precancerous lesions and genital warts. The implementation of an HPV vaccine in low- to middle-income countries has significant financial challenges [118].

Despite significant advancements in various HPV treatments, there is still a pressing need to create new, effective therapeutics for the carcinogenesis caused by HPV [119]. CRISPR/Cas9-based gene therapy for HPV infection is now a reality due to recent technological advancements. To damage the HPV genome, multiple studies have thus far described anti-HPV applications of the CRISPR/Cas9 system [119]. According to the findings, the CRISPR/Cas9 strategy offers a great deal of potential for advancement as a clinically useful treatment for disorders linked to HPV [120]. There are various HPV life cycle editing targets for CRISPR/Cas9. To enhance the therapeutic effects, CRISPR-related technologies still need to be developed [14].

### 5.2. Hepatitis B Virus (HBV)

HBV is still a health problem, as seen by the 350–400 million chronic HBV carriers estimated worldwide [14,121]. The family Hepadnaviridae is seen in people with persistent HBV infection. The hepatitis virus is a hepatotropic DNA virus that can lead to liver cancer and cirrhosis. It replicates by reverse transcription in host hepatocytes at the stage of RNA intermediates [108]. Eight genotypes (A–H) of the HBV genome have been determined taxonomically, and between any two of these, there are over 8% nucleotide variations [122]. Given the low likelihood of sustained viral response (SVR) or cure in HBV-infected individuals, novel and more potent HBV treatment regimens must be developed [103]. A novel method for the anticipation and dealing of HBV infectious illnesses may be possible given CRISPR/Cas9 technology’s rapid development [123]. Gene therapies currently offer the great ability for entering clinical submissions after incapacitating several methodological obstacles and have emerged as a promising prospective treatment for HBV infections, particularly in efficiently targeting cccDNA [124]. Two research teams separately reported suppressing HBV infection in preclinical applications using the gene-editing tools ZFNs or TALENs [125]. Lin et al. first looked into the ground-breaking effort to employ the CRISPR/Cas9 system in preventing HBV infection in vitro and in vivo in 2014 [126]. To successfully suppress viral replication and production, some studies have used specially engineered Cas9/sgRNA (or Cas9/multiplex gRNA) combinations to alter just one locus, which is often in the conserved region of the HBV genome For the objective of eradicating HBV genomes, several other research studies associated with the combination of CRISPR/Cas9 and other techniques (such as various chemicals or inhibitory systems) have also been established [127,128,129,130] A Cas9 variation known as dead Cas9 (dCas9) has also been shown to prevent HBV replication without removing the HBV genome [130,131]. A study was recently carried out to investigate NU7026, a powerful NHEJ inhibitor, which blocked the deprivation of cccDNA-mediated cleavages by CRISPR/Cas9. This study offers a mechanism for confirming the role of CRISPR/Cas9 in eradicating the HBV genome [132]. Similar to ZFNs and TALENs, there is a risk of viral escape mutants when CRISPR/Cas9 systems are used therapeutically in HBV-infected cells [133]. Despite this, nucleic acid editing techniques have the potential to cause desired alterations on the target DNA [14,134].

### 5.3. Human Immunodeficiency Virus (HIV)

Antiretroviral therapy has decelerated the spread of HIV and significantly improved the clinical outcomes connected with this viral infection. The transcriptionally silent but replication-competent provirus survives in a long-lived cell reservoir primarily constituted of memory CD4+ T cells, making a complete cure for HIV infection challenging. This reservoir is highly robust and resistant to antiviral medications as well as immune response effects, posing a considerable hurdle to the complete eradication of HIV infection [135]. HIV, a significant global disease that mostly consists of HIV-1 and HIV-2, calls for cutting-edge treatments [136]. New infections occur every day, according to a recent UNAIDS report. HIV-1 differs from HIV-2 in that it is more transmissible and harmful in the human host [137]. The so-called chronic sickness of AIDS eventually develops as a result of significant CD4+ T-cell depletion brought on by active HIV-1 replication in living organisms [138]. These HIV therapies, however, intended to block different viral life cycle stages [139], are nonetheless unable to cure the illness since HIV-1 has been permanently incorporated into the host DNA. In light of these findings, scientists have concentrated on treating AIDS using CRISPR/Cas9-based gene editing techniques to open up a wide range of new opportunities for HIV-1 prevention and treatment [140]. When a patient tests positive for HIV, highly active antiretroviral therapy (HAART) is usually started as soon as possible. It is made up of three or more antiretroviral drugs taken together. HAART is also known as antiretroviral therapy (ART) and combination antiretroviral therapy (CART). A key component of HAART is the simultaneous administration of several drugs that inhibit viral replication through different mechanisms. This prevents the spread of a virus that has developed resistance to one of the drugs through the combined action of the other two drugs. The Infectious Diseases Society of America describes HAART regimen management as a multifaceted procedure that should be carried out by or in consultation with a practitioner with specific expertise [141,142,143,144]. It should be carried out by or in consultation with a doctor with particular experience, according to the definition of the HIV-Medicine Association of the Infectious Diseases Society of America.

Nucleoside/nucleotide reverse transcriptase inhibitors (NRTIs), which are the most often prescribed medications for ART, are known to cause fatal lactic acidosis and peripheral neuropathy as a result of mitochondrial toxicity. Several NRTIs can also cause anaemia, lipodystrophy, and bone marrow suppression as unwanted side effects [145,146,147,148,149]. Tenofovir is often well tolerated; however, it can damage the kidneys or lower bone mineral density. Patients may consider other medications if they have a history of osteoporosis or renal impairment (eGFR less than 60 mL/min/1.73 m). It will be required to monitor hepatic function clinically and in the lab because the discontinuation of tenofovir formulations could cause an immediate deterioration of HBV. After using abacavir, patients with the HLA-B*5701 mutations are more likely to develop a CD8-mediated hypersensitivity reaction. Didanosine is rarely used since it can cause hepatomegaly and pancreatitis. Numerous research studies using CRISPR/Cas9 technology as a means for treating HIV/AIDS have been created quickly since the first two CRISPR/Cas9-based applications in the prevention of HIV-1 were reported by Cho and Ebina, respectively, in 2013 [12,150]. Targeting viral genomes and host genes has previously been two crucial strategies for battling HIV-1 infection. Despite the development of Cas9/multiplexed-sgRNA technology, there are yet no studies that specifically and jointly target the two coreceptor genes CCR5 and CXCR4 using CRISPR/Cas9 molecular scissors. The largest barrier to effective HIV infection control at the moment is the purging of latent viral reservoirs. Latent viral reservoirs, which mostly attach to dormant memory CD4+ T cells, can persist for up to 60 years, as seen in HIV patients taking ART therapy [151]. Scientifically speaking, stem cell transplantation (SCT) is not a recommended treatment for HIV/AIDS [152]. These two case reports indicate that SCT was first intended to treat cancer, not HIV/AIDS, in the two individuals. The accidental therapies give hope for the future use of customized gene therapy in the treatment of AIDS [153].

### 5.4. Herpes Simplex Virus (HSV)

HSV-1, frequently known as human herpesvirus-1, is the original participant in the family of human herpesviruses [154]. The HSV-1 genome has double-stranded DNA that is similar to other herpesviruses [155]. The first work utilizing the CRISPR/Cas9 expertise in contrast to HSV-1 prolific infection in cell culture was published by Roehm et al. Three diverse sections of the viral DNA that encode the “HSV-1 *ICP0*” protein were chosen as the targets for the three guide RNAs. ICP0 is a crucial HSV-1 immediate early (IE) regulatory protein that has a big impact on the expression and replication of the viral genes [154,156]. According to these findings, *ICP0* was inactivated by Cas9/gRNA in cells expressing functional gRNA and Cas9, as seen by the cells’ sharp reductions in the ability to maintain Δ*ICP0* HSV-1 multiplication [154]. It was discovered that *ICP0* antiviral action that disrupts PML bodies is interfered with by mutations brought on by Cas9/Grna [155]. Studies using fluorescence microscopy and biochemical methods revealed that HSV-1 infection and replication were suppressed, as well as ICP0 protein production being inhibited. Plaque assays revealed that Cas9 and gRNA-expressing cells had lower virus titres and proliferation [154]. The TC620 cells’ capacity to advance through the cell cycle, undergo apoptosis, or remain viable was unaffected by the appearance of Cas9 and gRNAs [155]. The anti-HSV-1 Cas9/gRNA systems had little off-target effects, according to SURVEYOR assays and PCR sequencing analysis, since no indel mutations were discovered in many representative human genes identified by bioinformatics screening investigations [154]. These findings imply that there is little cytotoxicity or off-target activity in the Cas9/gRNA system. After being transfected with HSV-1-eGFP, which had a GFP expression cassette, Vero cells were first treated with anti-HSV-1 gRNAs. GFP expression was then analysed as a sign of HSV-1 infection and replication [157]. The majority of gRNAs that targeted crucial HSV-1 genes effectively reduced viral replication [157]. Although the nonessential genes are targeted, this can largely be explained by the fact that the Cas9/gRNA system produced double-strand DNA breaks, rendering these genomes inactive for the formation of viral offspring [155]. The ability of nine gRNAs to alter the topmost three anticipated off-target locations in the human genome was examined to evaluate possible off-target editing by the CRISPR/Cas9 system. These 27 human genome loci were amplified by PCR and sequenced using DNA samples taken from gRNA-expressing and control cells. These loci showed no evidence of CRISPR/Cas9-induced editing, indicating that CRISPR/Cas9-mediated genome editing did not take place at undesirable locations [157]. When HSV-1 was reactivated in the cultured cell model harbouring functional gRNAs, replication was suppressed [157]. These findings align with those of a recent study that used the CRISPR/Cas9 systems to combat HSV-1 lytic infection in Vero cells [158].

### 5.5. Severe Acute Respiratory Syndrome Coronavirus 2 (SARS-CoV-2)

Numerous techniques, including RT-qPCR, sequencing-based techniques, and immunological procedures, have been used to diagnose SARS-CoV-2. Common methods of detecting SARS-CoV2 (RT-PCR, serology) have been restricted because of low accuracy and sensitivity of sample preparation, reagents, equipment, and various types of clinical specimens; consequently, more research is needed to find low-cost methods with high sensitivity to detect SARS-CoV2 [159]. The most recent CRISPR/Cas technology can therefore be employed to develop diagnostic or therapeutic procedures. The emergence of novel SARS-CoV-2 strains, highly contagious viruses, and asymptomatic individuals enable the disease to spread throughout the world, wreaking havoc on the healthcare system and the global economy [160]. Therefore, it is highly recommended that quick and precise diagnostic techniques be used to identify infected people and confine them in order to stop the deadly virus’s cycle of global spread. The most well-known genome editing technique, the CRISPR/Cas system, ushers in a new era in SARS-CoV-2 detection. CRISPR/Cas-based methods have high specificity and sensitivity without the need for expensive equipment, in contrast to conventional laboratory methods for detecting COVID-19, such as RT-qPCR and next-generation sequencing (NGS), which demand highly skilled technicians and expensive facilities, and serological tests that recognise antibodies specific to SARS-CoV-2 in later stages of infection. CRISPR/Cas-based methods would be ideal for simple tests that are crucial for the diagnosis of COVID-19 because of their high precision, specificity, portability, and minimal equipment requirements, particularly in developing nations or locations with a higher risk of infection, such as airports, ports, and emergency rooms [161,162,163,164,165].

## 6. Utilizing CRISPR/Cas9 Technology to Fight Viral Infections in Plants

Plants including herbs and crops are susceptible to a number of viral infections that can cause significant economic losses [166,167]. CRISPR/Cas9 technology manipulates plant viral defence mechanisms by recognising and deleting pathogenic genes that infiltrate them. It can also be used to develop agricultural cultivars with increased tolerance to several plant viruses [168]. The use of association genetics in plant breeding, with an emphasis on single nucleotide polymorphisms (SNPs) and other widespread molecular markers, has increased and produced vital high-throughput data for the detection of quantitative trait loci (QTLs). Utilizing primary resistance genes placed into cultivars with enhanced agronomic characteristics, the principal QTL in crop variety has been used to provide quantitative resistance to plant viruses [169]. CRISPR/Cas9 technology has effectively been used to generate virus-resistant crop cultivars and enables the generation of a wider spectrum of CRISPR variations suitable for many applications. However, one of the most popular uses of the CRISPR/Cas9 technology is gene disruption [170], which aids in overcoming the error-prone behaviour of cellular NHEJ (DNA-repair machinery). A frameshift mutation and gene function disruption are brought about by the insertion or deletion (InDel) of nucleotides at sgRNA-targeted locations [171]. By altering the function of the vulnerable gene(s), which changes the plant–virus interaction and reduces viral fitness in the host plant, this technique has been used to engineer virus resistance.

Since viral infections develop quickly and dynamically, managing viral diseases is difficult. By producing viral and non-viral proteins, host resistance (R) genes, and gene silencing via RNA interference, a number of researchers have significantly contributed to the development of resistant plants [172]. The benefits of CRISPR have significantly contributed to the development of plants resistant to DNA and RNA viruses. *A. thaliana* and *N. benthamiana* were used in the first experiment to develop CRISPR-mediated viral resistance against *yellow dwarf virus* (BeYDV) and *beet severe curly top virus* (BSCTV). Increased resistance to several *geminiviruses* was seen in tobacco when *yellow dwarf virus* (YDV) gRNAs coding for replication and cell mobility were overexpressed [173]. *Endogenous banana streak virus* (eBSV), a double-stranded DNA *badnavirus* that is a member of the *Caulimoviridae* family and which inhabits Musa spp., was rendered inactive by the expression of sgRNA that was designed to target the eBSV coding sequence using the CRISPR/Cas9 tool. In comparison to unmodified control plants, the transgenic banana plants exhibited mild symptoms as well as eBSV resistance [174]. The single-stranded RNA (ssRNA) viral genomes were successfully edited using CRISPR/Cas tools, including programmable RNA-guided RNPs such as FnCas9 and CRISPR/Cas13a (LshCas13a; a nuclease from Leptotrichia shahii) [175,176]. Cas13 offers new promise for eradicating dangerous plant viruses because most plant viruses have RNA genomes. FnCas9, a different Cas9 nuclease from *Francisella novicida*, interferes with plant translation and replication by targeting endogenous RNA [175,177]. CRISPR-mediated immunity against the viruses *cucumber mosaic virus* (CMV) and *tobacco mosaic virus* (TMV) was developed by expressing gRNAs and FnCas9 in *N. benthamiana* and *A. thaliana*, and these plants were observed with a significant reduction in virus accumulation and minimal symptoms. CRISPR/Cas13a-induced genome editing of *tobacco Potyvirus* and *Turnip Mosaic Virus* (TuMV) resulted in the development of immunity [176]. Resistance developed in tobacco and *Arabidopsis* following CRISPR-mediated editing of the *pea early browning virus* (PEBV) and *Tobacco rattle virus* (TRV) gRNAs [178]. The Cas13a/sgRNA-expressing transgenic potato plants conferred control of various Potato virus Y (PVY) strains and decreased disease symptoms in potatoes [179]. The LshCas13a system was used to create rice resistant to the *Southern rice black-streaked dwarf virus* (SRBSDV) and *Rice stripe mosaic virus* (RSMV) [180]. The benefit of Cas13 for specifically targeting RNA viral genomes in plants needs more research [181]. These research studies show that CRISPR/Cas-mediated targeted viral genome editing is a potent strategy for conferring viral disease resistance in plants.

## 7. Pros and Cons of CRISPR-Based Diagnosis Systems

For the correct treatment of emerging viral diseases, they must be diagnosed. Majorly, we have two different kinds of tests available to detect the viruses appropriately: antibody-based or serological tests and viral genome-based tests. Serological tests generally sense the existence of immunoglobulins in the patient’s blood, which is a sign of an active adaptive immune reaction against one or more viral infections in the body at that particular time [105,126,182]. Viral genome-based tests that are commonly based on qRT-PCR increase the copy number of RNA molecules by the process of reverse transcription. They are used to check the attendance of viral infection from the sample directly even before symptom generation. These tests are worthwhile over immunoglobulin-based tests to check the blowout viral infection [10,25]

Some drawbacks allied with these approaches have led scientists to develop DETECTR and SHERLOCK assays, which are CRISPR-based assays. DETECTR targets DNA, which is comparatively quicker and as exact as qRT–PCR, whereas SHERLOCK targets RNA [75,152]. The limitations for qRT–PCR tests include the unobtainability of individual shielding gear, genome taking out kits and chemicals, as well as sample collection and RNA isolation and purification ways and means. These challenges are also valid for CRISPR-based diagnostics in the initial steps. Some other leads of CRISPR/Cas in comparison with qRT–PCR comprise prompt turnaround time, isothermal gesture strengthening that avoids the prerequisite of thermos cycler, individual specific nucleotide selection and targeting, no necessity for multifaceted research laboratory arrangement, and incorporation with reachable reporting setups comprising horizontal stream bands [183].

CRISPR technology also has some loopholes which need to be improved. The limitations start with the nonspecific base pairing of sgRNA with the targeted cellular or acellular being’s genome. This process is termed an off-target phenomenon. The consequences of the off-target phenomenon include irregular cell signalling and incorrect interpretation of the findings. One more problematic situation that could be confronted while executing CRISPR-based genome modification procedures that tie to the objective sequence may be inhibited by the formation of a secondary structure and the RNA-associated proteins there [103,183]. Being a fast, efficient diagnostic approach, CRISPR/Cas can detect almost all the mutated versions of corona variants. Thus, this tool can be used for the epidemiological survey and in designing appropriate cure strategies so that contagious and infectious diseases could be prevented from rapidly spreading in the communities [183]. This could also identify infection in asymptomatic people. The leading minuses of the current testing indicate that they are unable to spot the virus instantly after septicity and prerequisite time to the escalation of the viral inoculum load [103]. As in the case of COVID infection, viral burden generally changes throughout the day and at dissimilar steps of septicity. Consequently, a qRT–PCR assessment might show false negative at the time when the viral burden is little, but it does not eliminate contagion, and hence, a more precise investigation is obligatory [161]. One of the most important strengths of the CRISPR/Cas diagnosis is its gRNA selection, which is taken from the conserved genomic locus among the variants of viruses. This strategy provides this tool sensitivity, as it can detect even novel mutated strains of viruses [102,103]. CRISPR/Cas-based ultramodern tools such as SHERLOCK (RNA detecting) and DETECTR (DNA detecting) are well-chosen tools and are gaining a lot of attention by being quicker in comparison to basic or modified polymerase chain reactions, as they use isothermal amplification methods and DNA polymerases which have the ability to dislocate and relocate the DNA, refuting the denaturation procedure [64,184]. Overall, the above tests have the ability to adapt by utilizing the lateral flow dipsticks and by making massive thermos cycling, revealing tools needless. Compact turnaround time and narrow tool requests mark CRISPR diagnostics as progressively more operative laboratory gear for speedy diagnostic tests. To make moveable, quick investigations appropriate and feasible for patient location, DETECTR can be appended with microfluidic/surface plasmon resonance mediated recognition arrangements, which is lacking in former tests, comprising qRT-PCR, which prerequisite costly arrangement [184]. CRISPR/Cas is also incapable in multiplexing. Pathogen-definite crRNAs can be programmed from conserved loci of the pathogen genetic material. The ability of multiplex diagnostics can be used to differentiate various pathogenic viruses or even diverse serotypes of the viral entity at the same time and from the unchanged sample [84,172]. CRISPR/Cas-based kits comprising different Cas proteins and a range of sgRNA with changed constructions and molecules for not the same resolutions are predictable to develop more prevalent tools. In general, it is anticipated that an optimistic future lies in wait for CRISPR/Cas-based diagnoses and molecular biology techniques [185].

## 8. Bioethical Issues

CRISPR/Cas9, as a gene-altering technology, is prevailing and has the potential to bring new strains or breeds of crops and animals, and it is capable in human disease diagnosis and treatment. However, it might lead to misapplication and ill use if in the wrong hands, such as alteration of germline or zygotic genomes. Reasonable bioethical worries have risen over time by many specialists. Such kinds of chromosome editing approaches have every possible potential to alter the human race dynamically, and our forthcoming human race may thus be maltreated [185]. Major concerns other than illegal germline mutation, are morality, eugenics to assist the fittest to stay alive, the promising upsurge of genetic copies or clones, modified or manipulated babies, and perchance super humans [186]. There is a cumulative worry and fear among scientists, security agencies, and other bioethics-concerned groups that the current speedy uprising in biological tools and techniques has pronounced chances to be abused in violent biotic weaponries curricula [186].

## 9. Conclusions and Outlook

The progress and precipitous development in the field of CRISPR/Cas9 have covered a vast area and now have become a fascinating field of biotechnology and empowering elementary research by providing several applications in the field of genetic engineering. Although we have gained a lot of knowledge in this field, many key questions have yet to be resolved, especially about the phenomenon of spacer incorporation. The reason behind recurrent lateral transfer of CRISPR locus and its regulatory mechanism also needs to be enlightened. Curiosity also surrounds the significant relationship between interference and adaptation in the course of primed spacer retention. Other topics such as the role of CRISPR in the non-immunity phenomenon, its ecological impact, countermeasures, etc., deserve further attention. The enormous use of CRISPR/Cas9 and continuous efforts to improve its editing capabilities ensure its full potential to serve society and could be proven as a real asset for the existing period of medicine. To use CRISPR/Cas in the therapy of different types of cancer, a number of clinical trials are currently under process. The sustained movement to improve and introduce novel approaches to deliver genetically engineered appliances inside the cells and to execute these tools for various therapeutic aspects will almost certainly allow us to see some prodigious pharmaceutical application of CRISPR/Cas methods in the diagnosis and treatment of emerging viral infections, genetic disorders, and cancer, which we are not able to treat at the present time.

## Data Availability

Data sharing does not apply to this article, as no datasets were generated or analysed during the current study.
